# Ethnic Inequalities in Healthcare Use and Care Quality among People with Multiple Long-Term Health Conditions Living in the United Kingdom: A Systematic Review and Narrative Synthesis

**DOI:** 10.3390/ijerph182312599

**Published:** 2021-11-29

**Authors:** Brenda Hayanga, Mai Stafford, Laia Bécares

**Affiliations:** 1 School of Education and Social Work, University of Sussex, Essex House, Falmer, Brighton BN1 9RH, UK; l.becares@sussex.ac.uk; 2 The Health Foundation, 8 Salisbury Square, London EC4Y 8AP, UK; mai.stafford@health.org.uk

**Keywords:** ethnic inequalities, healthcare use, care quality, multiple long-term conditions, UK

## Abstract

Indicative evidence suggests that the prevalence of multiple long-term conditions (i.e., conditions that cannot be cured but can be managed with medication and other treatments) may be higher in people from minoritised ethnic groups when compared to people from the White majority population. Some studies also suggest that there are ethnic inequalities in healthcare use and care quality among people with multiple long-term conditions (MLTCs). The aims of this review are to (1) identify and describe the literature that reports on ethnicity and healthcare use and care quality among people with MLTCs in the UK and (2) examine how healthcare use and/or care quality for people with MLTCs compares across ethnic groups. We registered the protocol on PROSPERO (CRD42020220702). We searched the following databases up to December 2020: ASSIA, Cochrane Library, EMBASE, MEDLINE, PsycINFO, PubMed, ScienceDirect, Scopus, and Web of Science core collection. Reference lists of key articles were also hand-searched for relevant studies. The outcomes of interest were patterns of healthcare use and care quality among people with MLTCs for at least one minoritised ethnic group, compared to the White majority population in the UK. Two reviewers, L.B. and B.H., screened and extracted data from a random sample of studies (10%). B.H. independently screened and extracted data from the remaining studies. Of the 718 studies identified, 14 were eligible for inclusion. There was evidence indicating ethnic inequalities in disease management and emergency admissions among people with MLTCs in the five studies that counted more than two long-term conditions. Compared to their White counterparts, Black and Asian children and young people had higher rates of emergency admissions. Black and South Asian people were found to have suboptimal disease management compared to other ethnic groups. The findings suggest that for some minoritised ethnic group people with MLTCs there may be inadequate initiatives for managing health conditions and/or a need for enhanced strategies to reduce ethnic inequalities in healthcare. However, the few studies identified focused on a variety of conditions across different domains of healthcare use, and many of these studies used broad ethnic group categories. As such, further research focusing on MLTCs and using expanded ethnic categories in data collection is needed.

## 1. Introduction

Long-term conditions (also known as chronic conditions) are health conditions that are currently uncurable and consequently are managed with medication and other therapies (e.g., cardiovascular disease, diabetes and depression) [1,2]. In the UK, it is estimated that between 23% and 27% of the population live with two or more long-term conditions, and this number is expected to rise in the coming decades [2,3,4]. These trends present a challenge not only for individuals but also for society and entire healthcare systems [5,6]. People with multiple long-term conditions (MLTCs) are more likely to have increased disability, poorer functioning, reduced well-being, lower quality of life and higher mortality [6,7]. The relationship between MLTCs and increased healthcare costs is well documented [8]. Further, the challenges in providing high quality care for people with MLTCs are recognized [9]. People with MLTCs have increased exposure to healthcare services and systems, which are often fragmented and/or tailored towards managing single health conditions, thereby hindering the holistic management of MLTCs [7]. This uncoordinated care may lead to extra obligations for patients and healthcare staff, threats to patient safety and an increase in patient-level frustration [10,11].

This study focuses on people from minoritised ethnic groups with MLTCs and how their patterns of healthcare use and care quality vary from their White counterparts. In line with other studies, we use the term minoritised ethnic group to refer to people who do not self-identify as belonging to the White majority ethnic group [12,13]. Commonly used acronyms such as BAME (Black, Asian and Minority Ethnic) can be exclusionary as they single out specific ethnic groups [14,15]. Other terms such as ‘minority’ can be associated with diminished status if we consider that, historically, the narrative of ‘minorities’ marked troubled histories of immigration control, policing, racial violence, inferiorisation and discrimination that were characteristic of daily life for early migrants to the UK from Africa, the Caribbean and Asia [16]. The term ‘minoritised’ places emphasis on how social positions are social constructions rather than practices and outcomes that are natural and inevitable [17].

There is some evidence to suggest that people from minoritised ethnic groups in the UK are at an increased risk of developing MLTCs when compared to the White majority population, and they are also more likely to develop MLTCs at an earlier age [18,19]. The findings from a recent review indicate a higher prevalence of MLTCs in some minoritised ethnic groups compared to their White counterparts [20]. These ethnic inequalities in MLTCs are likely to reflect broader economic and social inequalities, which in turn are driven by racism and racial discrimination [21,22]. These same mechanisms can lead to inequities in access and use of healthcare and care quality, which can lead to negative outcomes for people with MLTCs [23]. Studies of single conditions report that, in general, people from minoritised ethnic groups are less likely to access specialist services and less likely to report positive experiences of primary care when compared to their White counterparts [24,25,26]. It is possible that people with MLTCs from minoritised ethnic groups may face similar experiences when using healthcare services. Findings from a recent ethnographic study conducted by Revealing Reality for the Taskforce on Multiple Conditions give insight into how ethnic inequalities in healthcare use and care quality can arise [23]. The study explored the lives of people with MLTCs experiencing health inequity and disadvantage, living in some of the most deprived wards in the UK. This study illustrated how wider societal processes (e.g., deprivation and suboptimal healthcare provision) intersect with individual level processes (e.g., poor literacy skills, language difficulties, competing priorities) to negatively impact people’s ability to access and utilise healthcare services, adhere to treatment regimens and ultimately manage their MLTCs [23].

Whilst the aforementioned study gives insight into the experiences of people with MLTCs, including those from minoritised ethnic groups, their focus was not on uncovering ethnic inequalities. It is important to examine ethnic variations in healthcare use and healthcare quality among people with MLTCs. Findings of such an exploration can illuminate ethnic inequalities and inform actions to redress the health disadvantage faced by particular populations [27], which, if ignored, can result in the widening of existent ethnic inequalities. Given the increasing ethnic diversity of the UK population [28], a detailed examination of the association between MLTCs, healthcare and ethnicity in the UK is warranted.

Past reviews of healthcare use and care quality, which have included studies reporting on differences across ethnic groups, have focused on a particular domain of healthcare (e.g., access to healthcare [29]) or health services for a particular group of conditions (e.g., somatic healthcare service related to screening, general practitioners, specialists, emergency rooms and hospital care [30]). In one review, the authors synthesised the best evidence for improving healthcare quality for people from minoritised ethnic groups [31]. However, the focus of these reviews was not on people with MLTCs [29,30,31]. To our knowledge, no review has synthesised evidence on ethnic inequalities in healthcare use and care quality among people with MLTCs living in the UK. Such an undertaking can highlight areas where inequalities are evident and inform discussions and efforts to address them. Therefore, the aims of this review are (1) to identify and describe the literature that reports on ethnicity and healthcare use and care quality among people with MLTCs living in the UK and (2) to examine how healthcare use and/or care quality for people with MLTCs compares across ethnic groups in studies counting more than two long-term conditions.

## 2. Methods

### 2.1. Search Strategy

In line with the Preferred Reporting Items for Systematic review and Meta-Analysis Protocols (PRISMA-P) [32], we registered the protocol for this review on PROSPERO (CRD42020220702). Between October and December 2020, we searched the following databases for studies that compared healthcare use and/or care quality across different ethnic groups of people with MLTCs living in the UK: ASSIA, Cochrane Library, EMBASE, MEDLINE, PsycINFO, PubMed, ScienceDirect, Scopus and Web of Science core collection. We also conducted a search on OpenGrey to ensure that relevant grey literature was not excluded. We supplemented the electronic search with a manual search of the key studies identified. We contacted relevant authors when full texts were not available.

We followed the conventions of each search engine and used search terms that denoted the key concepts in this review: Ethnicity (e.g., “Ethnic Groups” [Mesh] OR “BME” OR “BAME”), Multiple health conditions (e.g., “Multiple Chronic Conditions” OR Comorbid* OR Multimorbidity), Health inequality (e.g., “Health Equity” [Mesh] OR “Healthcare disparit*” [MeSH] OR Inequalit*), Healthcare use (e.g., “Delivery of Healthcare” [Mesh] OR “Tertiary Healthcare” [Mesh]), Care quality (e.g., “Quality of Healthcare” [Mesh] OR “Patient Acceptance of Healthcare” [Mesh] OR “Patient Satisfaction” [Mesh]) and the geographical location (e.g., “United Kingdom” [MeSH Terms] OR “UK”) (See Appendix A for a full list of search terms).

### 2.2. Selection Criteria

We did not restrict the start of the search to any particular period in time and included only UK studies, published in English, reporting on healthcare use and/or care quality among people with MLTCs, across different ethnic groups of people living in the UK [33]. Our justification for focusing on studies in the UK was driven by the recognition that the UK has a unique healthcare system that is publicly funded, with a range of comprehensive services that are (mostly) free at the point of use [34]. Further, it has a diverse minoritised ethnic group population [35]. These factors would complicate comparisons with other countries with different healthcare, political, and economic systems and population structures.

In the extant literature, MLTCs are defined and operationalised in different ways. Some use the term MLTCs synonymously with the term multimorbidity (here defined as the presence of two or more long-term health conditions [3,36]). Others also incorporate the term comorbidity (i.e., the presence of any distinct additional co-existing ailment in an individual with an index condition under investigation [37,38]). Given these definitions, we included studies that counted only two conditions (e.g., diabetes and depression) as well as those that counted two or more long-term conditions. However, to address the second aim we excluded studies that counted only two conditions and focused on those that also counted more than two long-term conditions as they are more likely to give insight into those with complex medical needs and greater use of healthcare [39,40].

Healthcare use and care quality are broad concepts that encapsulate different domains. Healthcare use can be defined as the quantification or description of the use of services by persons for the purpose of preventing and curing health problems, promoting maintenance of health and well-being, or obtaining information about one’s health status and prognosis [41]. Indicators of healthcare use include GP consultations, hospital visits including inpatient, outpatient and day visits, hospital admissions, accidents and emergency department visits, diagnoses, prescriptions, referrals, immunisations and screening [29,42,43]. In contrast, healthcare quality has been defined as the degree to which healthcare services increase the chances of desired health outcomes for people and are aligned with current professional knowledge [44]. Indicators of care quality include effectiveness, patient-centeredness, efficiency, equity of care and principles such as acceptability, trust, responsiveness, safety, waiting times, patient experience, satisfaction with accessibility, humaneness of care, number of readmissions and cultural appropriateness [45,46]. We included studies regardless of the domain of healthcare use and care quality under investigation.

We imported the studies retrieved from the electronic search to Endnote X8. We first removed the duplicates. Following this, B.H. and L.B. screened a random sample (10%) of the titles and abstracts. Differences were resolved through discussion. B.H. proceeded to independently screen the remaining studies. The same process was repeated when screening the full texts.

### 2.3. Data Extraction

B.H. and L.B. extracted data from a random sample (10%) of the studies identified. Disagreements were settled by discussion. B.H. independently extracted data from the remaining studies. We extracted relevant information from the included studies using a structured form, which included the following items: study identifier, study design, geographical location, data source, sample size, population characteristics (e.g., age and gender profile, ethnic group categories), type and number of MLTCs, confounding variables and healthcare use and care quality domains and results.

### 2.4. Outcomes

The outcomes of interest were patterns of healthcare use and care quality among people with MLTCs for at least one minoritised ethnic group, compared to the White majority population.

### 2.5. Data Analysis

Owing to the lack of a common definition of healthcare use and care quality, the different domains of healthcare use and care quality assessed, the variety of conditions explored and the different ethnic group categories assessed in the included studies, we conducted a narrative synthesis of the findings. We present the findings of the synthesis in themes, and supplement the reporting with tables and figures. The findings are presented in two sections. First, we provide an overview of the studies that report healthcare use and care quality across ethnic groups of people with MLTCs, including the participant characteristics, domains of healthcare and care quality assessed, and types of health conditions under investigation. Second, we present the evidence of ethnic inequalities in healthcare use among people with MLTCs from the studies that went beyond counting only two long-term conditions. We use the terminology used by authors to describe ethnic categories in their studies.

## 3. Results

### 3.1. Overview of Included Studies

**Figure 1:**
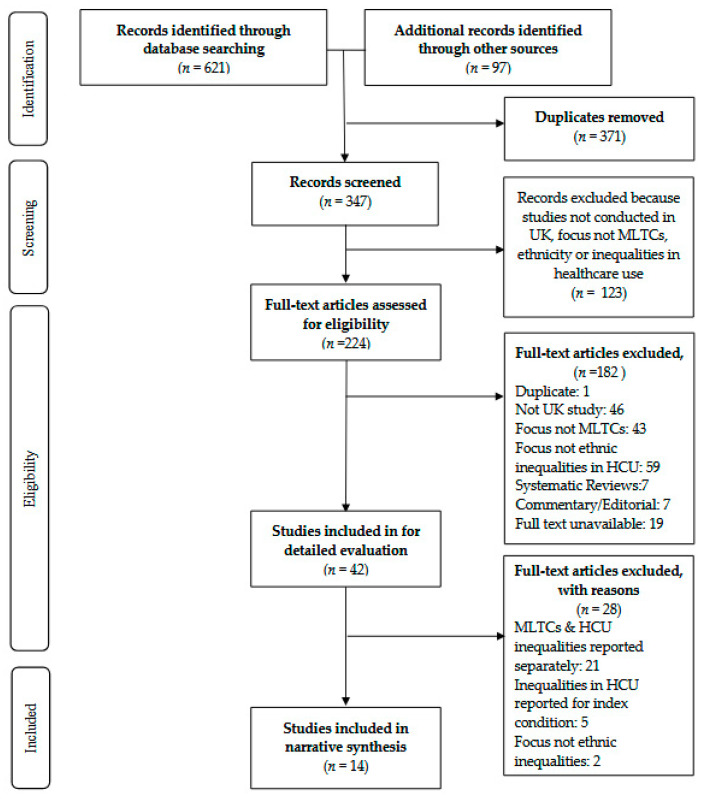
PRISMA flowchart [47].

We identified 621 titles from the electronic search (See Figure 1, which is based on PRISMA guidelines [47]). After removal of duplicates and studies identified as ineligible from the title or abstract, 42 papers were eligible for further evaluation. A further 28 studies were excluded because, despite reporting on the key concepts of interest (i.e., MLTCS, ethnicity, healthcare use), some reported MLTCs and healthcare use separately (*n* = 21), others reported inequalities in healthcare for one health condition (*n* = 5) and others did not compare healthcare use across the different ethnic groups (*n* = 2). Consequently, 14 studies were included in the review, with five of these studies contributing to the evidence on ethnic inequalities in healthcare use in people with MLTCs living in the UK. These were studies in which the authors counted more than two long-term conditions and not just two conditions. The former are more likely to illuminate patterns of ethnic inequality among those with complex medical needs and greater use of healthcare [39,40].

The 14 studies included in this review were published between 2001 and 2021. There were three national studies [48,49,50] and 11 local studies conducted in Birmingham [51], Leicester [52] and London [53,54,55,56,57,58,59,60]. The number of participants in the included studies ranged from 45 to nearly 61.5 million. The majority of studies used patient records. In eight of the 14 studies, data from primary care records were analysed [48,54,56,57,58,59,60,61]. The remaining studies used hospital records (*n* = 2) [49,50] and records from specialist services such as Diabetes Outpatient Clinics (*n* = 2) [52,55]. One study used data from the Comorbidity Dual Diagnosis Study [53], and another used data from a community-based Mental Health and Substance Misuse services survey [51].

### 3.2. Participant Characteristics

#### 3.2.1. Ethnic Group Identification

Nine of the 14 included studies explicitly reported how ethnicity was identified (64%). Of these, participants self-reported their ethnic identity in seven studies [53,54,55,56,57,60,61]. In one study, ethnicity was assigned by keyworkers [51], and in another study, computerised name recognition software was used to identify South Asian people [52].

#### 3.2.2. Ethnic Group Categorisation

Of the 14 included studies, two compared ethnic variations in healthcare use among people with MLTCs between two ethnic group categories. Of these studies, White people were compared to Black [58] and South Asian people [52]. Three studies categorised their participants into three ethnic group categories [54,55,56], and two studies compared outcomes across four ethnic groups [53,57]. The remaining studies grouped their participants into five or more ethnic group categories (*n* = 7) [48,49,50,51,59,60,61].

#### 3.2.3. Missing Ethnicity Data

Information concerning missing ethnicity data was available in nine of the 14 included studies (64%). In two of these studies, those with missing ethnicity data were labelled as missing/unknown and included in the analyses [48,49]. In the remaining seven studies, participants with missing ethnicity data were excluded from the analyses [51,54,55,56,57,59,60]. One study excluded participants who were of ‘Other’ ethnicity due to the heterogeneity by ethnicity within the group [60]. Only one study conducted sensitivity analyses to ascertain if the results would differ if those with missing ethnicity data were excluded [48].

#### 3.2.4. Gender and Age

There were 11 studies that reported the gender profile of the participants. One study included only female participants [61], and the remaining ten studies included both male and female participants [48,51,52,53,54,55,56,58,59,60]. Of the 14 included studies, six reported the mean age and standard deviation (SD). The average age of participants in these studies ranged from 26.8 (SD = 5.9) years to 66 (SD = 8.5) years [48,52,53,55,60,61]. Four studies included participants aged 18 years and above [51,54,57,59]. In one study, participants were aged 25 years and above [56], and in another, they were aged 16 years and above. [58]. The focus of one study was on children and young people aged between 10 years and 24 years [49], while another study included participants aged 10 years and over [50].

### 3.3. Domains of Healthcare Use and Care Quality Assessed in Included Studies

Table 1 below lists the domains and sub-domains of healthcare use and care quality assessed in the included studies. The most frequently assessed domain was disease management/monitoring (*n* = 6). Of these studies, the authors examined ethnic differences in diabetes management and cardiovascular risk factors monitoring among people with MLTCs. These were measured by assessing HbA1c levels, cholesterol levels, smoking status, protein urea levels and Body Mass Index, [52,54,55,57,58,60]. One study assessed ethnic differences in health screening, including mammography and cervical smears, among people with psychosis and comorbidities [58]. There were three studies that reported on ethnic differences in prescriptions among people with MLTCs [55,57,61]. Another three studies reported on the use of hospital services, including admission and length of hospital stay [49,50,53]. Few studies looked at disease progression (*n* = 2), mortality/risk of mortality (*n* = 2) and quality of treatment (*n* = 2). One study assessed the use of Mental Health and Substance Misuse services among people with severe mental health problems who use substances problematically [51].

### 3.4. Studies Reporting on Ethnic Differences in Patterns of Healthcare Use and Care Quality among People with Multiple Long-Term Conditions Living in the UK

Of the 14 included studies, 12 studies (86%) specified an index condition when reporting on ethnic differences in healthcare use and care quality among people with MLTCs (Table 2). The most frequently cited index conditions were diabetes (*n* = 6) [48,52,54,55,56,60] and mental health conditions (*n* = 4) [51,53,58,61]. One study focused on people with hypertension [59], and another assessed alcohol-related conditions as a comorbidity [50].

Two studies (14%) did not specify an index condition when examining ethnic inequalities in patterns of healthcare use and care quality among people with MLTCs. Of these studies, one assessed risk factor management among people with cardiovascular multimorbidity [57], while the other assessed emergency admissions and long-term conditions in children and young people [49].

### 3.5. Evidence of Ethnic Inequalities in Healthcare Use among People with Multiple Long-Term Conditions

In this review, five studies also counted more than two long-term conditions and are likely to give us insight into people with complex healthcare needs and greater use of healthcare [39,40]. Four studies focused on disease management, and one study focused on use of hospital services, in particular, emergency admissions. It would be inappropriate to combine their results because the studies represent different domains of healthcare use. Consequently, we discuss these two domains separately in the following section.

#### 3.5.1. Ethnic Inequalities in Disease Management among People with Multiple Long-Term Conditions

The four studies that suggest that there are ethnic inequalities across different domains of disease management among people with MLTCs are local studies that analysed data from primary care records using a cross-sectional study design, where the authors assessed the outcomes at a single point in time [52,54,57,58]. The sample sizes ranged from 1090 participants to 6690 participants, and comparisons were made between White participants and Black [54,57,58], South Asian [52,54,57], Asian [49] and those who self-identified as belonging to Mixed [49,57] and ‘Other’ ethnic groups [57]. Three of these studies specified an index condition: diabetes [52,54] and psychosis [58]. Mehta and colleagues (2011) assessed the relationship between glycaemic control, chronic disease comorbidity and ethnicity in people with diabetes. They found that among patients with Type 2 diabetes mellitus, the excess odds of having suboptimal glycaemic control (HbA1c ≥ 7%) was 1.86 (95% CI: 1.49 to 2.32) for South Asians, with a comorbidity relative to White Europeans. Taking into consideration cardiac disease comorbidity and non-cardiac disease comorbidity, South Asians (compared to White Europeans) with Type 2 diabetes had an excess risk of having suboptimal glycaemic control, with odds ratios of 1.91 (95% CI: 1.49 to 2.44) and 2.27 (95% CI: 1.50 to 3.43), respectively.

Alshamsan and colleagues (2011) set out to examine ethnic inequalities in diabetes management among people with and without comorbid health conditions after a period of sustained investment in quality improvement in the UK [54]. After adjusting for age, sex, diabetes duration, BMI, socioeconomic status and practice level clustering, they found that the presence of two or more cardiovascular comorbidities was associated with similar blood pressure control among White people and South Asian patients when compared with White people without comorbidity [54]. The mean difference in systolic blood pressure was +1.5 mmHg (95% Confidence Interval (CI): −0.3–3.3) and +1.4 mmHg (95% CI: −0.8–3.6), respectively [54]. In contrast, the presence of two or more cardiovascular comorbidities was associated with worse blood pressure control among Black patients, with a mean difference in systolic blood pressure of +6.2 mmHg (95% CI: 3.5–8.5) [54].

Similarly, Mathur and colleagues (2011) investigated the likelihood of reaching clinical targets for blood pressure, total serum cholesterol and glycated haemoglobin by ethnic group for patients with MLTCs [57]. Their results show that after adjusting for age, sex and clustering by general practice, among those with three to five cardiovascular morbidities, Black patients were less likely to meet their blood pressure target, with adjusted odds ratios (AORs) of 0.63 (95% CI: 0.53 to 0.75) [57]. However, there were no differences apparent between White and South Asian patients [57]. Among those with three to five morbidities, both South Asian and Black patients were less likely to reach an HbA1c target of ≤7.5% compared to White patients, with adjusted odds ratios of 0.69 (95% CI: 0.60 to 0.79) and 0.79 (95% CI: 0.67 to 0.93), respectively [57]. For total serum cholesterol in patients with three to five morbidities, South Asian patients were consistently more likely to reach the target of ≤4 mmol/L than patients of White ethnicity, with adjusted odds ratios of 1.65 (95% CI: 1.49 to 1.83), but Black patients were less likely to meet the cholesterol target (AOR: 0.83 (95% CI: 0.71 to 0.97)) [57]. Patterns in statin prescribing mirrored those for control of total cholesterol; compared to White patients, South Asian patients were more likely to be prescribed statin, but Black patients were less likely to be prescribed statin [57].

The findings from Pinto and colleagues (2010) also point to ethnic inequalities in disease management in people with MLTCs. They investigated ethnic differences in the primary care management of patients with psychosis and analysed health screening and monitoring rates according to the presence of comorbidity [58]. After adjusting for age and area-level deprivation, no significant differences were evident between White and Black patients in relation to cholesterol tests, blood pressure reading, BMI, smoking status and mammogram screening rates [58]. However, they found lower cervical smear rates in Black women with previously abnormal cervical smears, with an odds ratio of 0.22 (95% CI: 0.07–0.69) [58].

#### 3.5.2. Ethnic Inequalities in Emergency Admission among People with Multiple Long-Term Conditions

The findings of one study are suggestive of ethnic inequalities in hospital admissions, in particular, emergency admissions, in people with MLTCs [49]. The study conducted by Wijlaars and colleagues (2018) was a national cross-sectional study that used hospital records. The 763,199 children and young people who took part in this study were categorised into the following ethnic groups: White, Black, Asian, Mixed and Unknown [49]. The authors set out to explore whether changes in emergency admission rates during transition from paediatric to adult hospital services differed in children and young people (aged between 10 and 24 years) with and without underlying long-term conditions [49]. They considered emergency admission to be a clinically important indicator of poor health, which might be affected by the quality of healthcare received from the community during transition [49]. They excluded pregnancy-related admissions and injury-related admissions, with the exception of intentional self-harm, which could signify an underlying mental health condition [49]. After adjusting for age, sex, deprivation and transition, Black and Asian ethnicity were associated with an increase in emergency admission rates for children and young people with LTCs (Incidence Rate Ratio (IRR): 2.49, 99% CI: 2.39 to 2.60)) and Asian ethnicity (IRR: 1.13, 99% CI: 1.08 to 1.19) [49]. This study also found that across the whole sample, the rates of emergency admission increased at the age when young people transition from paediatric care to adult healthcare [49].

## 4. Discussion

### 4.1. Summary of Findings

Of the studies that counted more than two long-term conditions, there were no studies that reported on care quality and few explored ethnic inequalities in healthcare use among people with MLTCs. The findings from these few studies indicate that there are ethnic inequalities in emergency admission and some aspects of disease management among people with MLTCs. Both Asian and Black children and young people with MLTCs were more likely to have higher rates of emergency admissions when compared to their White counterparts [49]. The findings also suggest that some minoritised ethnic groups with MLTCs are at particular risk of suboptimal disease management. In particular, Black people with MLTCs were found to be less likely to be prescribed statins and to reach set targets for blood pressure, HbA1c levels and total serum cholesterol levels when compared to other ethnic groups [54,57]. In addition, Black women with MLTCs and previously abnormal smears had lower cervical smear rates compared to White women [58]. In contrast, South Asian patients with MLTCs were more likely to have better control of their blood pressure and total serum cholesterol, but less likely to meet targets for HbA1c levels when compared to patients with MLTCs from other ethnic groups [52,54,57]. However, given the few studies identified, the different domains of healthcare use under investigation and the different health conditions explored, our conclusions are tentative.

### 4.2. Comparison with Other Reviews

To our knowledge, this is the first review of studies reporting on ethnic inequalities in these domains of healthcare use among people with MLTCs in the UK. Therefore, it is difficult to make comparisons with other reviews that focus on different populations or particular dimensions of healthcare use. However, some of the findings of this review complement those of other reviews of ethnic inequalities in healthcare use that have not focused on MLTCs. For example, the evidence from a review conducted by Dixon-Woods and colleagues (2005) found that utilisation of primary care was generally high among most minoritised ethnic group populations, though there were important exceptions [29]. Just as in this review, they found that uptake of some preventative services (e.g., breast and cervical screening) was relatively lower for minoritised ethnic group people [29]. Their findings also suggest that there are important variations within and between minoritised ethnic groups in their utilisation of healthcare [29]. This variation was also evident in our review, as South Asian patients with MLTCs had better blood pressure and cholesterol control compared to Black patients with MLTCs [57].

### 4.3. Mechanisms

The association between MLTCs, socioeconomic status and healthcare use has been reported; people with MLTCs living in poverty have been found to be less likely to use health services than those with financial resources [62]. Given the close link between ethnicity and socioeconomic disadvantage [29], it is important to consider socio-economic disadvantage when interpreting ethnic inequalities in healthcare. Of the five studies that also counted more than two long-term health conditions, two adjusted for area-level deprivation and one adjusted for socioeconomic status (and other factors, e.g., age, sex and cardiovascular risk) [49,54,58]. Ethnic inequalities in disease management were still evident after adjustment of socio-economic deprivation (and other factors on the explanatory pathway), with Black people reported to have poorer disease management [54,58], and Black and Asian children more likely to have increased rates of emergency admission [49]. While Mathur and colleagues did not adjust for individual level deprivation, their analysis focused on populations living in the eight most socially deprived localities in Britain [57].

That ethnic inequalities for some groups still persisted after adjustment of deprivation (and other factors) in some of these studies suggests that the observed inequalities are likely to be driven by other factors. Given the complex, intersecting processes that shape the development of MLTCs and determine the use of healthcare and care quality [18,27], the mechanisms underlying the observed ethnic inequalities are likely to be the result of the interplay of several processes. Individual-level factors, such as poor management among some people [54] and cultural barriers to effective self-management [52], have been proposed as reasons underlying observed ethnic differences. However, we argue that understanding ethnic inequalities in healthcare use requires an appreciation of the ways in which individual-level processes (e.g., ethnicity and class) intersect with macrolevel processes (e.g., racism and discrimination) to produce inequalities [63]. International studies have illustrated how racism and negative discriminatory practices can result in mistrust of healthcare professionals and create barriers to compliance with treatment, timely diagnoses and treatment and healthcare use [64,65,66]. These processes can impact efforts to manage MLTCs among minoritised ethnic group populations, thereby resulting in ethnic inequalities. Further evidence is provided by Ben and colleagues (2017), who conducted a systematic review and meta-analysis of quantitative studies reporting on the associations between self-reported racism and different dimensions of healthcare service utilisation [67]. They found that people experiencing racism were approximately two to three times more likely to report reduced trust in healthcare systems and professionals, lower satisfaction with health services and perceived care quality, and compromised communication and relationships with healthcare providers [67]. As such, the influences of racism and discrimination cannot be ignored, as they directly and indirectly create conditions that disadvantage many from minoritised ethnic groups, which in turn can result in ethnic inequalities in healthcare use.

### 4.4. Strengths and Limitations

A limitation of this review is that a single reviewer initially screened the titles and abstracts and excluded irrelevant studies, which might have introduced a level of reviewer bias. It is therefore possible that we may have missed relevant studies [68]. However, a manual search of the reference list of key studies was conducted to increase the likelihood of identifying as many relevant studies as possible. In addition, a subset of studies (10%) were double-screened and extracted prior to the independent screening and extraction to reduce reviewer bias. While the interest in MLTCs and associations with healthcare utilisation, costs and healthcare systems has grown over the last decade [33], the guidelines to optimise care for people with MLTCs are fairly recent. For example, in 2016, the National Institute for Health and Care Excellence published guidance for healthcare professionals, people with MLTCs and their families/carers [69]. Thus, there has not been much time to assess care quality among people with MLTCs, and thus studies in this area are sparse. Those that have done so have not explored ethnic inequalities in care quality [70,71]. As such, they were not included in this review. Relatedly, there were no qualitative studies that met the inclusion criteria; therefore, the findings of this review are based on the evidence from quantitative studies. It is important to remember that evidence from qualitative studies is equally important as it gives us an in-depth understanding of the experiences of people with MLTCs while illuminating the processes that can lead to inequalities in healthcare use and care quality as reported above [23]. The findings from these studies can help healthcare systems adapt to the needs of people with MLTCs, thereby improving their health [72].

Despite these limitations, this review has several strengths. First, the review was informed by the PRISMA guidelines to facilitate the transparent reporting of the review process [47,73]. Second, we conducted the electronic search across a range of databases to locate (un)published studies and hand-searched the reference lists of relevant studies and systematic reviews to reduce the likelihood of missing key studies. Third, when synthesising the results of studies that contributed to the evidence of ethnic inequalities in healthcare use and care quality among people with MLTCs, we only included studies that also counted more than two long-term conditions to give us insight into ethnic inequalities in healthcare use among people with complex healthcare needs [40].

This review also highlighted the limitations of the studies conducted in this area. For example, the review has illuminated the limited range of long-term conditions considered. The majority of studies included in this review focused on index conditions, particularly diabetes [52,54,56], mental health conditions [51,53,58,61] and cardiovascular disease [59]. As such, we have a partial understanding of ethnic inequalities in healthcare use among people with MLTCs. In addition, many of these studies categorised their participants into broad ethnic categories. In the five studies that contributed to the evidence of ethnic inequalities in healthcare use among people with MLTCs, minoritised ethnic group people were often clustered into Black [49,54,57,58], South Asian [52,54,57], Asian [49], Mixed [49,57] and Other [57] ethnic categories. It is important to note that in certain circumstances, combining individual ethnic groups into larger categories can facilitate the identification of broad patterns, given that some may have shared experiences of racism, discrimination, marginalisation and social exclusion [53]. However, these broad ethnic categories may mask the extent of intra-ethnic inequalities. For example, as reported above, Black people with MLTCs may be at particular risk of poor disease management [54,57,58]. However, the Black ethnic group population is diverse, and healthcare use and care quality might vary among the different subgroups. Findings from Afuwape and colleagues (2006) exemplify this notion [53]. They examined the characteristics of a community cohort with psychosis and comorbid substance misuse by ethnic group and found that Black Caribbean people had the longest mean contact with mental health services compared to Black African, Black Other and White patients [53]. This study highlights the value of disaggregating broad ethnic group categories. This nuanced approach is more likely to lead to the identification of those who are most vulnerable to developing MLTCs and in greatest need of intervention, and moves away from essentialising minoritised populations.

It is likely that reported ethnic inequalities are underestimated. The studies that contributed to evidence of ethnic inequalities in disease management and emergency admission all analysed data from patient records from primary and secondary care. Ethnicity recording across the National Health Service has improved markedly over the past decade [74]. However, there is evidence that ethnicity coding for patients who self-identify as White British is recorded correctly, but there are higher levels of incorrect coding of the ethnicity of patients from minoritised ethnic groups [75]. Others have also found that in most cases, hospital records over-represent ‘Other’ ethnic group categories while under-representing ‘Mixed’ ethnic groups and some specific ethnic groups [76]. Incomplete or inaccurate recording of ethnicity data makes it difficult to reliably assess health needs, access and outcomes across different ethnic groups [76]. Many of the studies included in this review excluded people in the ‘Other’ ethnic group. It is therefore possible that these studies underestimate the true extent of ethnic inequalities in emergency admission and disease management among people with MLTCs.

### 4.5. Implications

The observed inequalities in disease management across ethnic groups suggest that universal coverage and investment in quality initiatives may not be adequate and that enhanced strategies or targeted interventions are needed to improve equity of disease management across populations [52,54]. It is possible that the observed ethnic inequalities in emergency admission among children and young people with MLTCs from minoritised ethnic groups might not only be due to a higher level of ill health but also the poor management of health conditions in primary care. This finding also suggests that ethnic inequalities in healthcare use and care quality start early in the life course. However, further research is required to unpack these findings.

As mentioned previously, the minoritised ethnic group population in the UK is diverse and consists of those born outside the UK and those born in the UK [28]. With different migration histories, the length of residence in the UK among those born outside the UK will vary and may impact healthcare utilisation. Interestingly, studies exploring the association between healthcare use and the number of years spent in the UK have found mixed evidence [77,78,79]. One study found no differences in healthcare use between non-UK-born migrants and the UK-born population [79]. Another reported that international migrants were less likely to have used secondary care than established residents and within-England migrants [77]. These findings mirror those of Saunders and colleagues (2021), who found that newly arrived migrants have lower healthcare utilisation levels than the UK-born population, a pattern partially explained by younger age and lower levels of ill health [78]. However, these studies do not explicitly focus on populations with MLTCs. Given that none of the studies included in the review considered length of residence in the UK, further research is required to ascertain whether there is an association between length of residence, healthcare use among people with MLTCs and observed ethnic inequalities reported in this review.

The limitations of the studies identified in this review reflect the methodological challenges of investigating ethnic inequalities in healthcare use and care quality among people with MLTCs [29]. Evidently, more work is required to develop a comprehensive understanding of the extent of ethnic inequalities in healthcare use and care quality among people with MLTCs living in the UK. Future studies would need to consider how best to address the challenge of varying definitions for healthcare use and care quality. They would need to include people with a range of MLTCs and include more ethnic group categories, including marginalised White populations (e.g., Gypsy, Roma and Traveller communities), who have been reported to have poor health outcomes when compared to people from other communities [80,81]. They would also need to assess ethnic variations in other domains of healthcare and account for both individual-level and area-level deprivation and how they intersect with other factors. Such studies would add to the sparse evidence base in this area and allow for national and international comparisons.

In this review, studies that counted more than two long-term conditions that reported on care quality were lacking. If we consider that the assessment of care quality among people with MLTCs is in its infancy, this finding is not surprising. However, future studies should also aim to explore ethnic inequalities in care quality. Studies that adopt a longitudinal approach to analysing ethnic inequalities in healthcare use and care quality are required. These studies would give insight into the longitudinal association of MLTCs, healthcare use and care quality delivered with health outcomes across different ethnic groups [7,27]. Future studies would also benefit from conceptualising and analysing ethnic inequalities in healthcare use and care quality in people with MLTCs through an intersectional lens that considers the complex, multifaceted processes [63] that lead to the development of MLTCs and influence healthcare use and care quality. Such work could illuminate the extent to which key explanatory pathways, including racism and discrimination, contribute to the development of ethnic inequalities. The findings of such analyses could inform discussions on how ethnic inequalities in healthcare use and care quality among people with MLTCs can be effectively addressed.

## 5. Conclusions

This review identified few studies reporting on ethnic inequalities in healthcare use among people with MLTCs living in the UK. It illustrates a sparse evidence base, characterised by studies focusing on different health conditions and different domains of healthcare, which precludes us from drawing any firm conclusions. Indeed, the few studies identified are suggestive of ethnic inequalities in emergency admissions and particular domains of disease management among people with MLTCs. However, the methodological limitations of the studies identified in this review hamper our understanding of the full extent of ethnic inequalities in healthcare use and care quality among people with MLTCs. Based on these limitations, we call for action and have provided directions for future studies that we hope will provide evidence that can inform targeted prevention and management strategies to reduce inequalities in healthcare use and care quality among people with MLTCs.

## Figures and Tables

**Table 1:** Domains and sub-domains of healthcare use and care quality assessed in included studies.

Domains of Healthcare Use/Care Quality	Number of Studies	Sub-Domains of Healthcare Use and Care Quality
Disease management/monitoring	6	▪ Monitoring glycaemic control/HbA1c levels [52,54,55,57,60] ▪ Monitoring of cholesterol levels [54,55,57,58] ▪ Monitoring of blood pressure levels [54,57,58] ▪ Monitoring of smoking habit [55,58] ▪ Body Mass Index [58] ▪ Monitoring of protein urea levels [55] ▪ Cervical smears [58] ▪ Mammography [58]
Prescriptions	3	▪ Statins [57] ▪ Anti-depressants and anxiolytics [61] ▪ ACE inhibitors, β-blockers, calcium channel blockers, α-blockers, diuretics [55]
Use of hospital services	3	▪ Emergency admission [49] ▪ Hospital admission [53] ▪ Alcohol-related admissions [50] ▪ Length of stay in hospital [53]
Mortality/Risk of Mortality	2	▪ In-hospital mortality [48] ▪ Risk of death [56]
Disease progression	2	▪ Rate of renal decline [55,56]
Treatment quality	2	▪ Incorrect treatment [59] ▪ Complex treatment [55]
Tertiary service utilisation	1	▪ Use of Mental Health and Substance Misuse services [51]

**Table 2:** Characteristics of included studies.

Study ID	Study Design	Geographical Location	Data Source	Sample Size	Participant Characteristics	Ethnic Group Categories	Number of Conditions	Index Condition	Sub-Domain of Healthcare	Covariates
Afuwape, 2006 [53]	Retrospective cohort	Local	Comorbidity Dual Diagnosis Study	213	%Female: 16; Mean Age: 37 years	White, Black Caribbean, Black African, Black British	2	Psychotic illness	Hospital admission, length of stay in hospital, service satisfaction	-
Barron, 2020 [48]	Cross-sectional	National	General practice records	61,414,470	%Female: 50.1; Mean Age (SD): 40.9 (23.2)	Asian, Black, Mixed, Other, White, Unknown	2	Diabetes,	In-hospital mortality	-
Barry, 2015 [50]	Cross-sectional	National	Hospital Episode Statistics	264,870	%Female: NR; Age: 10+ years	White British, White Irish, Black Caribbean, Black African, SA—Pakistani and Bangladeshi, SA—Indian	2	Alcohol-related health conditions	Hospital admissions	-
Das-Munshi, 2021 [60]	Longitudinal study	Local	Primary care records	56,770	%Female: 46; Mean Age (SD): 63 (14)	White British, Irish, Black African, Black Caribbean, Bangladeshi, Indian, Pakistani, Chinese	2	Diabetes	Glycaemic management	age, gender, deprivation
Earle, 2001 [55]	Retrospective case note review	Local	Diabetes Outpatient Clinic	45	%Female: 36; Mean Age (SD): 66 (8.5)	Indo-Asian, African-Caribbean, Caucasian	2	Diabetes	Systolic and diastolic blood pressure, glycaemic control, and usage of ACE inhibitors, β-blockers, calcium channel blockers, α-blockers, diuretics, rate of renal decline, antihypertensive regimen	-
Graham, 2001 [51]	Cross-sectional	Local	Community-based Mental Health andSubstance Misuse services	498	%Female: 22.2; Age: 18+ years	White UK, African-Caribbean, Asian, European, Irish, Mixed race, Black other, Other	2	Severe mental illness	Use of Mental Health and Substance Misuse services	-
Mathur, 2018 [56]	Observational community-based cohort study with nested case–control	Local	General practice records	99,648	%Female: 56; Age: 25+ years	White, South Asian, Black	2	Diabetes	Rate of decline, and risk of death	age, sex and baseline measures of HbA1c, eGFR, CVD, ACE/ARB and diabetes duration
Prady, 2016 [61]	Cross-sectional	Local	Primary care records	2234	%Female: 100; Mean Age (SD): 26.8 (5.9)	White British, Pakistani, Mixed, Indian, White non-British, Black, Bangladeshi, Other	2	Common mental disorders	Drug prescription for common mental disorders	-
Schofield, 2012 [59]	Cross-sectional	Local	Lambeth DataNet	28,320	%Female: 50.9; Age: 18+ years	White, Mixed, Asian or Asian British, Black or Black British, Chinese or Other	2	Hypertension	NICE recommended treatment	-
Alshamsan, 2011 [54]	Cross-sectional	Local	Electronic medical records	6690	%Female: 49.1; Age: 18 years	White, Black, South Asian	10	Diabetes	Diabetes management (HbA1c, total cholesterol, and blood pressure levels)	age, sex, diabetes duration, BMI, socioeconomic status, and practice level clustering
Mathur, 2011 [57]	Cross-sectional	Local	Primary care records	6274	%Female: NR; Age: 18+ years	White, South Asian, Black, Other	5	-	Cardiovascular multimorbidity risk management, cholesterol, blood pressure, blood glucose levels HbA1c levels, statin prescriptions	age and sex, clustered by general practice
Mehta, 2011 [52]	Cross-sectional study	Local	Outpatient diabetes clinic	5664	%Female: 45.6; Mean Age (SD): 33 (13)	South Asian, White European	12	Diabetes	Diabetes management (glycaemic control)	-
Pinto, 2010 [58]	Cross-sectional study	Local	Lambeth DataNet	1090	%Female: 39.9; Age: 16+ years	White, Black	5	Psychosis	Health screening and chronic disease monitoring measures (record of cervical smears, mammograms, cholesterol testing, blood pressure readings and smoking status); BMI recorded	age and IMD-2004 score
Wijlaars, 2018 [49]	Cross-sectional study	National	Hospital Episode Statistics	763,199	%Female: NR; Age range: 10–24 years	White, Black, Asian, Mixed, Unknown	9	-	Emergency admission	age, sex, IMD, transition

ACE: Angiotensin-converting-enzyme inhibitors; ARB: Angiotensin II Receptor Blockers; BMI: Body Mass Index; CVD: Cardiovascular disease; eGFR: estimated glomerular filtration rate; HbA1c: Haemoglobin A1c; IMD: Index of Multiple Deprivation; NICE: National Institute for Health and Care Excellence; NR: Not reported; SD: Standard Deviation.

## Data Availability

This study did not report any supporting data.

## Appendix A

**Table A1:** Search terms used when searching Applied Social Sciences Index and Abstracts.

10	MLTCs + Ethnicity + inequality + quality care + country	(((MAINSUBJECT.EXACT.EXPLODE (“Mixed ethnicity”) OR MAINSUBJECT.EXACT. EXPLODE (“Ethnicity”) OR ab,ti,if (“Ethnic Group?” OR “african continental ancestry group” OR Arab OR Africa? OR Afro? OR Asian OR “Asian Continental Ancestry Group” OR “Asylum seeker” OR Bangladesh? OR Black OR “BME” OR “BAME” OR Caribbean OR China OR Chinese OR Cultur? OR Divers? OR Ethnic? OR Gypsy OR India? OR Irish OR Migrant OR Minorit? OR Mixed OR “Mixed ethnic?” OR “Multiple ethnic?” OR Multi rac? OR ‘Other White’ OR Pakistan? OR Roma OR “White Other” OR Refugee? OR race OR racial? OR “South Asian” OR “European Continental Ancestry Group”)) AND (MAINSUBJECT.EXACT (“England and Wales”) OR MAINSUBJECT.EXACT (“Channel Islands”) OR MAINSUBJECT.EXACT (“UK”) OR MAINSUBJECT.EXACT (“Scotland”) OR MAINSUBJECT.EXACT (“England”) OR MAINSUBJECT.EXACT (“Northern Ireland”) OR MAINSUBJECT.EXACT (“Wales”) OR ti, ab, if (“United Kingdom” OR “UK” OR England OR Wales OR Scotland OR “Northern Ireland” OR Britain OR “Great Britain”)) AND (ti, ab, if (“Multiple Chronic Conditions” OR Co morbid? OR Multi morbidity OR Multi patholog? OR “multiple condition?” OR “Multiple health condition?” OR “Multiple health problems” OR “Multiple medical conditions” OR “Multiple medical problems” OR “Pluri$patholog?” OR Polymorbid? OR “multiple illness?” OR “Multiple Chronic Health Conditions” OR “Multiple Chronic Medical Conditions” OR “multiple chronic illness?”) OR MAINSUBJECT.EXACT.EXPLODE (“Comorbidity”))) NOT (MAINSUBJECT.EXACT. EXPLODE (“USA”) OR MAINSUBJECT.EXACT.EXPLODE (“North America”) OR MAINSUBJECT.EXACT.EXPLODE (“Canada”) OR MAINSUBJECT.EXACT.EXPLODE (“Australia”) OR MAINSUBJECT.EXACT.EXPLODE (“New Zealand”) OR MAINSUBJECT.EXACT. EXPLODE (“South America”) OR MAINSUBJECT.EXACT.EXPLODE (“Central America”) OR ti, ab, if (“Americas” OR “USA” OR America OR “North America” OR Canada OR Australia OR “New Zealand”))) AND (ti, ab, if (“Health Equity” OR “Healthcare disparit?” OR Inequalit? OR disparit? OR “Healthcare Disparit?” OR “Health care Disparit?” OR “Health-care Disparit?” OR “Health Care Inequalit?” “Healthcare Inequalit?” OR “Health-care Inequalit?” OR “inequalit? in healthcare” OR “inequalit? in health care” OR “inequality in health-care” OR “disparit? in healthcare” OR “disparit? in health care” OR “disparit? in health-care” OR “inequit?” OR “health inequit?”) OR MAINSUBJECT.EXACT (“Health inequalities”)) AND (ti, ab, if (“Quality of Health Care” OR “Patient Acceptance of Health Care” OR “Patient Satisfaction” OR “Health Care Quality, Access, and Evaluation” OR “Care Quality” OR “Quality of care” OR “Quality of health care” OR “quality of health-care” OR “Quality of healthcare” “healthcare quality” OR “health-care quality” OR “health care quality” OR “quality health service” OR “health service quality” OR satisfaction OR dissatisfaction OR satisfied OR dissatisfied OR “effectiveness” OR safety OR responsiveness OR acceptab? OR appropriate? OR timeliness) OR MAINSUBJECT.EXACT. EXPLODE (“Quality of care”))
9	MLTCs + Ethnicity + Healthcare use + inequality + country	(((MAINSUBJECT.EXACT.EXPLODE (“Mixed ethnicity”) OR MAINSUBJECT.EXACT. EXPLODE (“Ethnicity”) OR ab,ti,if (“Ethnic Group?” OR “african continental ancestry group” OR Arab OR Africa? OR Afro? OR Asian OR “Asian Continental Ancestry Group” OR “Asylum seeker” OR Bangladesh? OR Black OR “BME” OR “BAME” OR Caribbean OR China OR Chinese OR Cultur? OR Divers? OR Ethnic? OR Gypsy OR India? OR Irish OR Migrant OR Minorit? OR Mixed OR “Mixed ethnic?” OR “Multiple ethnic?” OR Multi rac? OR ‘Other White’ OR Pakistan? OR Roma OR “White Other” OR Refugee? OR race OR racial? OR “South Asian” OR “European Continental Ancestry Group”)) AND (MAINSUBJECT.EXACT (“England and Wales”) OR MAINSUBJECT.EXACT (“Channel Islands”) OR MAINSUBJECT.EXACT (“UK”) OR MAINSUBJECT.EXACT (“Scotland”) OR MAINSUBJECT.EXACT (“England”) OR MAINSUBJECT.EXACT (“Northern Ireland”) OR MAINSUBJECT.EXACT (“Wales”) OR ti, ab, if (“United Kingdom” OR “UK” OR England OR Wales OR Scotland OR “Northern Ireland” OR Britain OR “Great Britain”)) AND (ti, ab, if (“Multiple Chronic Conditions” OR Co morbid? OR Multi morbidity OR Multi patholog? OR “multiple condition?” OR “Multiple health condition?” OR “Multiple health problems” OR “Multiple medical conditions” OR “Multiple medical problems” OR “Pluri$patholog?” OR Polymorbid? OR “multiple illness?” OR “Multiple Chronic Health Conditions” OR “Multiple Chronic Medical Conditions” OR “multiple chronic illness?”) OR MAINSUBJECT.EXACT.EXPLODE (“Comorbidity”))) NOT (MAINSUBJECT.EXACT. EXPLODE (“USA”) OR MAINSUBJECT.EXACT.EXPLODE (“North America”) OR MAINSUBJECT.EXACT.EXPLODE (“Canada”) OR MAINSUBJECT.EXACT.EXPLODE (“Australia”) OR MAINSUBJECT.EXACT.EXPLODE (“New Zealand”) OR MAINSUBJECT.EXACT. EXPLODE (“South America”) OR MAINSUBJECT.EXACT.EXPLODE (“Central America”) OR ti, ab, if (“Americas” OR “USA” OR America OR “North America” OR Canada OR Australia OR “New Zealand”))) AND (ti, ab, if (“Health Equity” OR “Healthcare disparit?” OR Inequalit? OR disparit? OR “Healthcare Disparit?” OR “Health care Disparit?” OR “Health-care Disparit?” OR “Health Care Inequalit?” “Healthcare Inequalit?” OR “Health-care Inequalit?” OR “inequalit? in healthcare” OR “inequalit? in health care” OR “inequality in health-care” OR “disparit? in healthcare” OR “disparit? in health care” OR “disparit? in health-care” OR “inequit?” OR “health inequit?”) OR MAINSUBJECT.EXACT (“Health inequalities”)) AND (ti, ab, if (“Delivery of Health Care” OR “Tertiary Healthcare” OR “Primary Health Care” OR “Health Care Quality, Access, and Evaluation” [Mesh] OR “Community Health Services” OR Healthcare OR health-care OR “health care” OR “health service” OR “health centre” OR “Health centre” OR “medical care” OR “National Health Service” OR “NHS” OR A E OR “Accident and emergency” OR “Acute healthcare” OR “Acute health care” OR “Acute health-care” OR “Acute hospital care” OR “urgent care” OR “emergency care” OR “primary care” OR “general practitioner” OR “GP” OR “General pract? visit” OR “GP visit?” OR “GP consult?” OR “General pract? consult?” OR “medical consult?” “GP services” OR “General practitioner services” OR “physician visit” OR “Family Physician” OR Dental OR Dentist OR dentistry OR “Eye care” OR Optician OR “Oral health” OR Pharmacy OR pharmacies OR “pharmacy service” OR “Secondary care” OR Hospital OR “Hospital visit” OR “hospital admission” OR “Day patient” OR in-patient OR “inpatient” OR outpatient OR out-patient OR referral OR therap? OR “Preventative healthcare” OR “preventative health care” OR “preventative health-care” OR “preventative service” OR “preventative medicine” OR “health outreach” OR screen? OR vaccinat? OR “Palliative care” OR “Case manag?” OR “Community care” OR “Community nurse” OR “Community services?” OR “Tertiary care” OR “tertiary health care” OR “tertiary healthcare” OR “tertiary health-care” OR specialist OR “specialist health service” OR “Mental health service” OR “sexual health service”) OR MAINSUBJECT.EXACT.EXPLODE (“Health care”))
8	quality care	ti, ab, if (“Quality of Health Care” OR “Patient Acceptance of Health Care” OR “Patient Satisfaction” OR “Health Care Quality, Access, and Evaluation” OR “Care Quality” OR “Quality of care” OR “Quality of health care” OR “quality of health-care” OR “Quality of healthcare” “healthcare quality” OR “health-care quality” OR “health care quality” OR “quality health service” OR “health service quality” OR satisfaction OR dissatisfaction OR satisfied Or dissatisfied OR “effectiveness” OR safety OR responsiveness OR acceptab? OR appropriate? OR timeliness) OR MAINSUBJECT.EXACT.EXPLODE (“Quality of care”)
7	healthcare utilisation	ti, ab, if (“Delivery of Health Care” OR “Tertiary Healthcare” OR “Primary Health Care” OR “Health Care Quality, Access, and Evaluation” [Mesh] OR “Community Health Services” OR Healthcare OR health-care OR “health care” OR “health service” OR “health centre” OR “Health centre” OR “medical care” OR “National Health Service” OR “NHS” OR A&E OR “Accident and emergency” OR “Acute healthcare” OR “Acute health care” OR “Acute health-care” OR “Acute hospital care” OR “urgent care” OR “emergency care” OR “primary care” OR “general practitioner” OR “GP” OR “General pract? visit” OR “GP visit?” OR “GP consult?” OR “General pract? consult?” OR “medical consult?” “GP services” OR “General practitioner services” OR “physician visit” OR “Family Physician” OR Dental OR Dentist OR dentistry OR “Eye care” OR Optician OR “Oral health” OR Pharmacy OR pharmacies OR “pharmacy service” OR “Secondary care” OR Hospital OR “Hospital visit” OR “hospital admission” OR “Day patient” OR in-patient OR “inpatient” OR outpatient OR out-patient OR referral OR therap? OR “Preventative healthcare” OR “preventative health care” OR “preventative health-care“ OR “preventative service” OR “preventative medicine” OR “health outreach” OR screen? OR vaccinat? OR “Palliative care” OR “Case manag?” OR “Community care” OR “Community nurse” OR “Community services?” OR “Tertiary care” OR “tertiary health care” OR “tertiary healthcare” OR “tertiary health-care” OR specialist OR “specialist health service” OR “Mental health service” OR “sexual health service”) OR MAINSUBJECT.EXACT.EXPLODE (“Health care”)
6	Health inequality	ti, ab, if (“Health Equity” OR “Healthcare disparit?”OR Inequalit? OR disparit? OR “Healthcare Disparit?” OR “Health care Disparit?” OR “Health-care Disparit?” OR “Health Care Inequalit?” “Healthcare Inequalit?” OR “Health-care Inequalit?” OR “inequalit? in healthcare” OR “inequalit? in health care” OR “inequality in health-care” OR “disparit? in healthcare” OR “disparit? in health care” OR “disparit? in health-care” OR “inequit?” OR “health inequit?”) OR MAINSUBJECT.EXACT (“Health inequalities”)
5	MLTCs + Ethnicity + Country	(#1 AND #2 AND #3) NOT #4 ((MAINSUBJECT.EXACT.EXPLODE (“Mixed ethnicity”) OR MAINSUBJECT.EXACT.EXPLODE (“Ethnicity”) OR ab,ti,if (“Ethnic Group?” OR “african continental ancestry group” OR Arab OR Africa? OR Afro? OR Asian OR “Asian Continental Ancestry Group” OR “Asylum seeker” OR Bangladesh? OR Black OR “BME” OR “BAME” OR Caribbean OR China OR Chinese OR Cultur? OR Divers? OR Ethnic? OR Gypsy OR India? OR Irish OR Migrant OR Minorit? OR Mixed OR “Mixed ethnic?” OR “Multiple ethnic?” OR Multi$rac? OR ‘Other White’ OR Pakistan? OR Roma OR “White Other” OR Refugee? OR race OR racial? OR “South Asian” OR “European Continental Ancestry Group”)) AND (MAINSUBJECT. EXACT (“England and Wales”) OR MAINSUBJECT.EXACT (“Channel Islands”) OR MAINSUBJECT.EXACT (“UK”) OR MAINSUBJECT.EXACT (“Scotland”) OR MAINSUBJECT. EXACT (“England”) OR MAINSUBJECT.EXACT (“Northern Ireland”) OR MAINSUBJECT. EXACT (“Wales”) OR ti, ab, if (“United Kingdom” OR “UK” OR England OR Wales OR Scotland OR “Northern Ireland” OR Britain OR “Great Britain”)) AND (ti, ab, if (“Multiple Chronic Conditions” OR Co$morbid? OR Multi$morbidity OR Multi$patholog? OR “multiple condition?” OR “Multiple health condition?” OR “Multiple health problems” OR “Multiple medical conditions” OR “Multiple medical problems” OR “Pluri$patholog?” OR Polymorbid? OR “multiple illness?” OR “Multiple Chronic Health Conditions” or “Multiple Chronic Medical Conditions” OR “multiple chronic illness?”) OR MAINSUBJECT.EXACT.EXPLODE (“Comorbidity”))) NOT (MAINSUBJECT.EXACT.EXPLODE (“USA”) OR MAINSUBJECT.EXACT.EXPLODE (“North America”) OR MAINSUBJECT.EXACT.EXPLODE (“Canada”) OR MAINSUBJECT.EXACT. EXPLODE (“Australia”) OR MAINSUBJECT.EXACT.EXPLODE (“New Zealand”) OR MAINSUBJECT.EXACT.EXPLODE (“South America”) OR MAINSUBJECT.EXACT. EXPLODE (“Central America”) OR ti, ab, if (“Americas” OR “USA” OR America OR “North America” OR Canada OR Australia OR “New Zealand”))
4	excluded countries	MAINSUBJECT.EXACT.EXPLODE (“USA”) OR MAINSUBJECT.EXACT.EXPLODE (“North America”) OR MAINSUBJECT.EXACT.EXPLODE (“Canada”) OR MAINSUBJECT.EXACT. EXPLODE (“Australia”) OR MAINSUBJECT.EXACT.EXPLODE (“New Zealand”) OR MAINSUBJECT.EXACT.EXPLODE (“South America”) OR MAINSUBJECT.EXACT. EXPLODE (“Central America”) OR ti, ab, if (“Americas” OR “USA” OR America OR “North America” OR Canada OR Australia OR “New Zealand”)
3	Country	(MAINSUBJECT.EXACT (“England and Wales”) OR MAINSUBJECT.EXACT (“Channel Islands”) OR MAINSUBJECT.EXACT (“UK”) OR MAINSUBJECT.EXACT (“Scotland”) OR MAINSUBJECT.EXACT (“England”) OR MAINSUBJECT.EXACT (“Northern Ireland”) OR MAINSUBJECT.EXACT (“Wales”)) OR ti, ab, if (“United Kingdom” OR “UK” OR England OR Wales OR Scotland OR “Northern Ireland” OR Britain OR “Great Britain”)
2	Ethnicity	(MAINSUBJECT.EXACT.EXPLODE (“Mixed ethnicity”) OR MAINSUBJECT.EXACT. EXPLODE (“Ethnicity”)) OR ab,ti,if (“Ethnic Group?” OR “african continental ancestry group” OR Arab OR Africa? OR Afro? OR Asian OR “Asian Continental Ancestry Group” OR “Asylum seeker” OR Bangladesh? OR Black OR “BME” OR “BAME” OR Caribbean OR China OR Chinese OR Cultur? OR Divers? OR Ethnic? OR Gypsy OR India? OR Irish OR Migrant OR Minorit? OR Mixed OR “Mixed ethnic?” OR “Multiple ethnic?” OR Multi$rac? OR ‘Other White’ OR Pakistan? OR Roma OR “White Other” OR Refugee? OR race OR racial? OR “South Asian” OR “European Continental Ancestry Group”)
1	Multiple long-term conditions (MLTCs)	ti, ab, if (“Multiple Chronic Conditions” OR Co$morbid? OR Multi$morbidity OR Multi$patholog? OR “multiple condition?” OR “Multiple health condition?” OR “Multiple health problems” OR “Multiple medical conditions” OR “Multiple medical problems” OR “Pluri$patholog?” OR Polymorbid? OR “multiple illness?” OR “Multiple Chronic Health Conditions” or “Multiple Chronic Medical Conditions” OR “multiple chronic illness?”) OR MAINSUBJECT.EXACT.EXPLODE (“Comorbidity”)
